# Getting fit for hip and knee replacement: The Fit-Joints multimodal intervention for frail patients with osteoarthritis – a pilot randomized controlled trial

**DOI:** 10.1016/j.tjfa.2025.100028

**Published:** 2025-03-04

**Authors:** Chinenye Okpara, Ahmed Negm, Jonathan Derrick Adachi, David Armstrong, Stephanie Atkinson, Victoria Avram, Justin de Beer, Genevieve Hladysh, George Ioannidis, Courtney Kennedy, Patricia Hewston, Arthur Lau, Justin Lee, Julie Richardson, Sharon Marr, Akbar Panju, Danielle Petruccelli, Lehana Thabane, Mitchell Winemaker, Alexandra Papaioannou

**Affiliations:** a Department of Health Research Methodology, Evidence and Impact, McMaster University, Hamilton, ON, Canada; b Faculty of Rehabilitation Science, University of Alberta, Edmonton, AB, Canada; c Department of Medicine, McMaster University, Hamilton, ON, Canada; d Department of Pediatrics, McMaster University, Hamilton, ON, Canada; e Department of Surgery, Division of Orthopedic Surgery, McMaster University, Hamilton, Ontario, Canada; f The YMCA of Hamilton/Burlington/Brantford, Hamilton, ON, Canada; g School of Rehabilitation Science, McMaster University, Hamilton, ON, Canada; h Geras Centre for Aging Research, Hamilton Health Sciences, Hamilton, ON, Canada; i The Research Institute of St Joseph's Healthcare, Hamilton, ON, Canada; j Faculty of Health Sciences, University of Johannesburg, Johannesburg, South Africa

**Keywords:** Frailty, Joint replacement, Osteoarthritis, Rehabilitation, Older adults

## Abstract

**Background:**

Older adults with frailty have high risk for poor postoperative outcomes.

**Objective:**

To evaluate the feasibility of a multimodal prehabilitation program in older adults with frailty awaiting hip or knee replacement.

**Design:**

Parallel two-arm randomized controlled pilot trial.

**Participants and setting:**

Community-dwelling older adults with frailty awaiting joint replacement aged ≥60 years recruited from the Musculoskeletal Central Intake and Assessment Centre (MSK CIAC), Ontario.

**Intervention:**

Exercise, protein and vitamin D supplements, and medication review.

**Measurement:**

Feasibility was assessed based on predefined progression criteria for recruitment, retention, data completion and adherence to intervention components. Clinical outcomes including Oxford Knee and Hip Scores, frailty index, Short Physical Performance Battery and health-related quality of life were collected at baseline, 1-week preoperative, 6-weeks and 6-months postoperative and were evaluated using generalized linear mixed models for repeated measures.

**Results:**

A total of 69 participants were enrolled. Recruitment rate was 35 %. Participants’ mean age was 74 (standard deviation (SD): 7.5); 51 % were prefrail and 36 % were frail. Participant retention was 81 %, and data completion was ≥80 %. Mean adherence to strength exercises was 4 days (95 % confidence interval (CI): 3–5 days/week), balance 3 days (95 % CI: 2–4 days/week), and flexibility 3 days (95 % CI: 3–4 days/week). Adherence to vitamin D intake was 82 % (95 % CI: 73–92 %), and medication review consultation completion was 86 % (95 % CI: 68–95 %). These outcomes met the target values for feasibility success. The Oxford Knee Score at 6-months postoperative 8.78 (95 % CI: 0.40–17.16) showed a clinically meaningful and statistically significant difference between treatment groups. There were also indications of clinically relevant changes for frailty and quality of life post-surgery.

**Conclusion:**

This trial provides strong evidence of feasibility and indications of improvements in postoperative clinical outcomes. Challenges to implementation and adherence were identified that can inform modifications to study design for future trials.

**Trial registration:**

ClinicalTrials.gov NCT02885337. Registered August 31, 2016. https://classic.clinicaltrials.gov/ct2/show/NCT02885337

## Key messages on feasibility


 

• There are limited well-designed and generalizable trials on how to prehabilitate older adults with frailty scheduled for hip or knee replacement surgery to improve postoperative outcomes.
• The FitJoints pilot trial demonstrated that participant retention, data completion, adherence to strength, balance and flexibility exercises, adherence to vitamin D supplement intake and medication review consultation are feasible.
• There were also signals of benefits for knee pain, railty, and quality of life with the intervention.
• Future trials should consider strategies to improve participant recruitment and exercise accountability, protein supplements tailored to participant preference and current dietary needs, as well as measures to optimize the implementation of medication review recommendation.

## 1 Introduction

Joint replacement is one of the most common surgeries among older adults [1]. With the growing aging population, the demand for orthopedic surgical services is rising [2,3]. Recent estimates in Canada show approximately 110,000 hip and knee replacements were performed between 2020 and 2021, despite cases cancelled due to the COVID-19 pandemic [4]. Estimates from the USA indicate a projected rise in the number of total hip and knee arthroplasties of 71 and 85 % respectively by 2030 [3]. These major surgeries provide substantial improvements in pain, physical function, and quality of life for patients with osteoarthritis (OA) [5] . However, the presence of pre-existing conditions, such as frailty, may reduce the benefits of a joint replacement surgery, increase the risk of surgical complications, and impair recovery [6,7].

Frailty is characterized by low physiological reserve and decreased function arising from an accumulation of age and disease-related deficits [8,9]. Persons living with frailty have weakened resilience and increased vulnerability to stressors [8,9]. Therefore, the stress of a major surgery may induce the deterioration of physiological reserve, leading to poor surgical outcomes [6]. Frailty has been linked with increased risk of mortality, postoperative complications, prolonged length of stay, discharge to institutional care, functional decline, new disability, lower quality of life and discharge to institutional care following surgery [2,7,10,11]. In major, elective, non-cardiac surgeries including arthroplasty, healthcare costs and resource use are considerably higher in older adults with frailty [12] with a 1.5-fold increase in the cost of postoperative care in the year after surgery [13]. These issues underscore the need for preoperative interventions to manage frailty and minimize adverse postoperative outcomes in this population.

Multimodal interventions including physical exercise and nutrition supplementation have been shown to be beneficial in managing frailty [14] and may potentially lead to better surgical outcomes in patients with frailty [15]. Prehabilitation is a process consisting of at least one preoperative intervention (e.g., exercise, nutrition, psychological strategies) that can enhance the functional and adaptive capacity of patients with frailty to cope with upcoming stressors by improving physiological reserve [15,16]. Recent systematic reviews and meta-analyses examining the effectiveness of prehabilitation interventions in orthopedic surgical patients have reported varying conclusions regarding the preoperative and postoperative benefits [17,18]. In the frail general surgical population, the evidence supporting prehabilitation is also unclear [19]. Most studies evaluated in these reviews were among younger or healthier populations while studies specifically examining the high-risk population of older adults with frailty awaiting joint replacement are lacking. The few trials [20,21,22] available in this patient group have some methodological and interventional limitations to consider including, short duration of intervention (3 – 6 weeks), inclusion of hip replacement patients or females only, and assessment of short-term outcomes. These limitations have also been suggested in previous trials of prehabilitation in older adults with frailty undergoing cancer surgery [23,24]. A qualitative study also suggested that tailored prehabilitation programs may be important to improve adherence in people with frailty [25]. In other areas such as cardiac surgery, prehabilitation has demonstrated benefits for functional capacity, frailty, postoperative complications, and length of stay in frail patients [26]. More well-designed studies are essential to inform the perioperative management of these older patients for better health outcomes. As the first step to a larger definitive trial, we conducted a pilot study to assess the feasibility of implementing a multimodal intervention in older adults with frailty scheduled to undergo total hip or knee replacement.

## 2 Methods

### 2.1 Study design

The FitJoints pilot study is a two-arm, parallel group, randomized controlled trial (RCT) evaluating the feasibility and effectiveness of multimodal frailty intervention compared to usual care among prefrail/frail older patients awaiting elective total hip or knee replacement. The trial was registered with ClinicalTrials.gov NCT02885337. The reporting of this study adheres to the CONSORT extension for pilot and feasibility studies statement [27]. We provide a summary of the study design, setting, population, recruitment, intervention and control arm description, study outcomes, and data analysis. Full details of the study methods can be found in the published study protocol [28].

### 2.2 Study setting

The study was conducted between September 2016 and October 2019. Participants were recruited from Musculoskeletal Central Intake and Assessment Centre (MSK CIAC) formerly Regional Joint Assessment Program (RJAP) at Juravinski Hospital – a tertiary care hospital in Hamilton, Ontario, Canada. The MSK CIAC program caters to individuals with arthritis who are recommended by their primary care provider to be assessed for hip or knee replacement. This assessment is conducted by orthopedic surgeons and advanced practice physiotherapists (APPs) with specialized orthopedic training.

### 2.3 Study population and recruitment strategy

Inclusion criteria for participants were: (a) ≥ 60 years old; (b) prefrail (score of 1 – 2) or frail (score of 3 – 5) based on Fried frailty phenotype [9]; (c) awaiting elective unilateral hip or knee replacement; and (d) scheduled for a surgery with wait time between 3 – 10 months. Exclusion criteria included: self-reported renal insufficiency, neuromuscular disorder, active cancer, or any inflammatory arthritis. Orthopedic surgeons and APPs assessed patients referred for hip or knee surgery, then APPs invited potential participants deemed as surgical candidates and screened them for eligibility. Potential participants were given a study information sheet to guide their decision about participation and were later contacted by a research assistant (RA) to confirm participation. Those who indicated interest to participate provided written informed consent. Study staff randomized participants to the intervention or usual care group on a 1:1 ratio based on a computer-generated stratified block randomization list generated and kept securely by a research staff external to the study. The stratification was based on the orthopedic surgeon conducting the surgery, age, and joint type. Baseline assessments were conducted after randomization by blinded outcome assessors and the first intervention visit by the study kinesiologist was scheduled for the intervention group participants. Those blinded to the intervention were outcome assessors, data entry personnel, data analysts, clinical administrators who assigned participants’ surgery dates, the investigative team, and steering committee. Due to the nature of the study, it was not possible to blind other intervention personnel and participants.

### 2.4 Intervention development

The FitJoints intervention was designed to address the complex nature of frailty using multi-modal interventions as in the Australian FIT trial [29]. The components of the intervention were informed by existing evidence including a systematic review on exercise interventions in prefrail and frail older adults [30], protein supplement recommendations by guidelines [31,32], available evidence on vitamin D including a trial on oral vitamin D in a similar population conducted by the investigators of this study [33] and a meta-analysis that demonstrated benefits with vitamin D on muscle strength and balance in older adults [34], and explicit criteria and guidelines for medication appropriateness in older adults [35,36]. A team of researchers, healthcare providers and stakeholders met to discuss the relevance, application, and adaption of existing evidence to the study context and population.

### 2.5 Multimodal arm

Participants in the intervention group received a multimodal intervention comprising physical exercise, protein supplement and dietary counselling, vitamin D supplement for 3 – 10 months between randomization and surgery, as well as a one-time medication review consultation. The description of the intervention follows the TIDieR guideline [37].

**Exercise**: The study kinesiologist performed an introductory fitness assessment and goal setting with intervention participants at their homes based on the tools for assessing physical fitness of older adults by Jones and Rikli [38]. The kinesiologist developed tailored exercise programs for each participant and conducted bi-weekly appointments (i.e., one monthly home visit and one interim phone-call) to review participant progress and adjust exercise programs as required. The exercise prescription was based on the recommendations from the Canadian Physical Activity Guidelines for older adults ≥65 years [39] and included aerobic, strength, flexibility and balance components. Participants were encouraged to exercise 3 times/week for 45 – 60 mins [30] either at home or at a local YMCA (free membership provided for the duration of the intervention) with fitness and pool classes designed for individuals with joint issues of their preference. They were given a logbook to track their exercise activities which included frequency of balance, flexibility, and strength exercises, as well as frequency and duration of aerobic exercises.

**Protein supplement**: Participants received a commercially-available protein supplement containing 20*g* protein, 350 kcal and 1.5*g* β-Hydroxy β-Methylbutyrate/serving to be taken daily, with a meal on non-activity days, or within 3 h of exercise on activity days. The supplements were delivered by the kinesiologist during routine visit to participants who were also provided with a dietary intake log for tracking frequency of supplement consumption.

**Vitamin D**: All intervention participants were supplied with vitamin D3 (1000 IU/day tablets) to be taken once daily.

**Medication review**: The study pharmacist reviewed the medications of participants in the intervention group using the Screening Tool of Older Person's Prescription STOPP/Screening Tool to Alert to Right Treatment START criteria [35] and Beers criteria [36]. Based on this review, they provided written recommendations to the participant's family physician. Participants were encouraged to follow up with their family physician to review and implement the recommendations which were faxed or mailed to the physicians. The medication review consultation was conducted once per participant during the intervention period.

### 2.6 Control arm

Participants in the control group received usual care which may have included recommendations by their surgeon to attend a physical exercise program or improve fitness before surgery, discharge planning, and home safety information during a preoperative educational class. However, the study kinesiologist provided no additional fitness support or advice.

### 2.7 Study outcomes

**Feasibility outcomes:** The primary outcome was feasibility assessed by (1) recruitment rate (percentage of patients enrolled out of all patients eligible for hip or knee replacement), (2) retention rate (percentage of participants who completed intervention phase and completed the study) and (3) data completion (percentage of participants with complete clinical outcomes data at the 6 months postoperative timepoint). The predetermined criterion for progression was set at 80 % for all of these measures. Participant's self-reported adherence to each component of the intervention was also assessed as part of the feasibility outcomes. The exercise component with corresponding feasibility targets were (1) strength (≥2 days/week), (2) aerobic (≥150 mins/week), (3) balance (≥2 days/week), and (4) flexibility (≥2 days/week). The expected frequency or duration of the exercise types was based on the Canadian Physical Activity Guidelines for older adults ≥65 years [39]. Other aspects of the intervention were assessed as follows: protein supplement (percentage of daily protein supplements consumed), Vitamin D (percentage of daily Vitamin D supplements consumed), and medication review (percentage of participants who received medication review and the percentage who implemented the review recommendations). Adherence ≥80 % was considered adequate for the nutrition component.

**Clinical outcomes:** The clinical outcomes evaluated include: (1) Oxford Hip and Knee Score – patient-reported outcome used to assess functional ability and pain in patients undergoing total hip or knee replacement with high validity, reliability and responsiveness [40,41] (2), Frailty – assessed using the Geras Fit-Frailty app which is based on the frailty index of deficit accumulation [42] (3) Short Physical Performance Battery (SPPB) – validated tool for assessing functional mobility with good internal consistency and sensitivity to change [43], and (4) European Quality of Life 5 Dimension 3 Level (EQ-5D-3L) for the assessment of patient's health-related quality of life [44]. Higher scores for EQ-5D-3L (range: 0 – 1), Oxford Hip and Knee Scores (range: 0 – 48) and SPPB (range: 0 – 12) indicate better outcomes while higher scores for frailty index (range: 0 – 1) suggests poorer outcomes.

**Adverse events:** Adverse events were self-reported, unfavourable experiences during the study period. Based on the participant's report of the event, the research coordinator determined if the adverse event was related or unrelated to the intervention (e.g., occurred during prescribed exercises). If the research coordinator had any other concerns or was unsure about the report, they would communicate with the principal investigator. Fatal or life-threatening events were considered serious adverse events and were reported to the Research Ethics Board. An independent Data Safety and Monitoring Board reviewed the trial data for safety.

### 2.8 Data collection and management

Study outcomes were collected at baseline, 1 week preoperative, 6 weeks postoperative and 6 months postoperative by blinded assessors who were trained by the research coordinator. Trained research assistants conducted monthly visits to track intervention adherence and monthly phone check-ins to all participants to monitor adverse events. Study data were managed using REDCap database.

### 2.9 Data analysis

Participants baseline characteristics were summarised as means with standard deviation and frequencies with percentages for continuous and categorical variables respectively. The feasibility outcomes were analysed descriptively and presented as mean score with corresponding 95 % confidence interval (CI). The clinical outcomes were analysed on an intention-to-treat basis using generalized linear mixed-effects modeling for repeated measures and included time, treatment group, and time by treatment group interaction as independent variables. The estimated between-group treatment effects and associated 95 % CI were obtained for each of the three study follow-up visits. Per protocol analyses were also performed in the same way and were restricted to participants with complete data at all time points. All analyses were performed using Stata version 17 (StataCorp, College Station, TX).

### 2.10 Sample size estimation

The sample size was based on the feasibility outcomes of 80% (i.e., for screening, retention, data completion, and adherence with the intervention components). A sample size of 62 patients was estimated to produce a two-sided 95% confidence interval with a width equal to ± 10% for 80% adherence. The estimation was conducted using PASS software (Kaysville, Utah).

### 2.11 Ethical considerations

All participants provided signed written informed consent before enrollment. The study was approved by the Hamilton Integrated Research Ethics Board (file #2017 – 1565).

## 3 Results

### 3.1 Participant demographics

**Fig 1:**
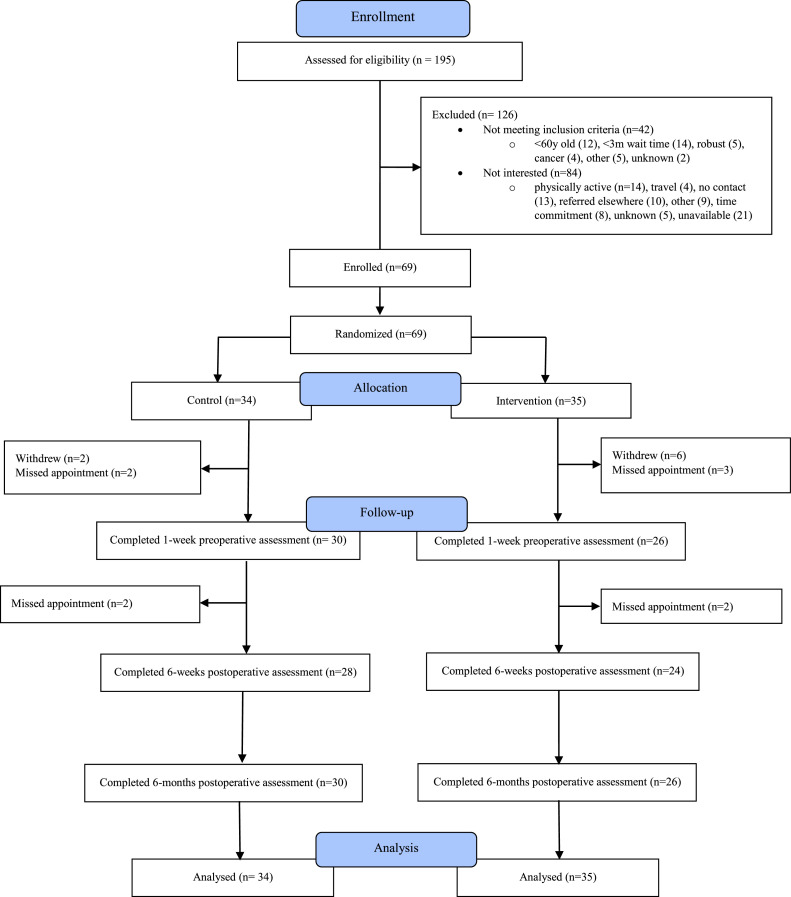
CONSORT flow chart of participants.

Participant enrolment occurred from September 2016 to January 2018. Fig. 1 shows the CONSORT flow chart of participants and Table 1 shows the baseline characteristics of participants by study arm. A total of 69 participants were enrolled and randomized: 34 in the control and 35 in the intervention arm. The mean age of the participants was 74 (SD: 7.5) of whom 51 % were prefrail and 36 % were frail, and 13 % were robust. The majority of the participants were female (68 %), had high school diploma or lower (61 %) and lived with others (i.e., family members including a partner, children, or siblings) (71 %). The mean Oxford Hip and Knee Scores were 20 (SD:7.3) and 21 (SD:7.7) respectively.

**Table 1:** Baseline Characteristics of Participants.

Characteristic	All	Control	Intervention
Age, mean (SD)	73.9 (7.5)	72.3 (7.6)	75.9 (7.1)
Age, n (%)			
<75	38 (55.1)	23 (67.7)	15 (42.9)
75 – 84	25 (36.2)	9 (26.5)	16 (45.7)
≥85	6 (8.7)	2 (5.9)	4 (11.4)
Sex, n (%)			
Male	22 (31.9)	12 (35.3)	10 (28.6)
Female	47 (68.1)	22 (64.7)	25 (71.4)
Body Mass Index, mean (SD)	33.9 (7.2)	30.3 (6.4)	33.5 (7.7)
Living arrangement n (%)			
Lives with others	49 (71.0)	25 (73.5)	24 (68.6)
Lives alone	20 (29.0)	9 (26.5)	11 (31.4)
Education n (%)			
≤High school	42 (60.9)	17 (50.0)	25 (71.4)
>High school	27 (39.1)	17 (50.0)	10 (39.1)
Smoking n (%)			
Former	31 (44.9)	11 (32.4)	20 (57.1)
Current	4 (5.8)	3 (8.8)	1 (2.9)
Never	34 (49.3)	20 (58.8)	14 (40.0)
Falls in the past year n (%)			
No	40 (58.0)	18 (52.9)	22 (62.9)
Yes	29 (42.0)	16 (47.1)	13 (37.1)
Walking aid use n (%)			
No	21 (30.4)	13 (38.2)	8 (22.9)
Yes	48 (69.6)	21 (61.8)	27 (77.1)
Previous fractures n (%)			
No	32 (46.4)	14 (41.2)	18 (51.4)
Yes	37 (53.6)	20 (58.8)	17 (48.6)
Comorbidity			
0	8 (11.6)	4 (11.8)	4 (11.4)
1	33 (47.8)	16 (47.0)	17 (48.6)
2 – 3	28 (40.6)	14 (41.2)	14 (40.0)
Oxford Hip Score, mean (SD)	19.5 (7.3)	20.0 (8.5)	19.1 (6.0)
Oxford Knee Score, mean (SD)	20.5 (7.7)	20.5 (8.6)	20.6 (7.3)
Frailty index, mean (SD)	0.30 (0.11)	0.30 (0.12)	0.29 (0.10)
Fried frailty phenotype			
Non-frail	9 (13.0)	7 (20.6)	2 (5.7)
Prefrail	35 (51.0)	17 (50.0)	18 (51.4)
Frail	25 (36.0)	10 (29.4)	15 (42.9)
EQ-5D-3L, mean (SD)	0.68 (0.11)	0.68 (0.12)	0.69 (0.11)
SARC-F, mean (SD)	4.1 (2.31)	4.1 (2.43)	4.2 (2.22)
SPPB, mean (SD)	6.77 (2.31)	6.88 (2.45)	6.66 (2.19)
No of medications			
<5	17 (24.6)	9 (26.5)	8 (22.9)
≥5	52 (75.4)	25 (73.5)	27 (77.1)

SD, standard deviation; n, number; %, percentage; EQ-5D-3L, European Quality of Life,5 Dimension 3 Levels;.

SARC-F, Strength, Assistance with walking, Rising from a chair, and falls; SPPB, Short Physical Performance Battery.

### 3.2 Feasibility

The feasibility results are shown in Table 2. A total of 1017 patients were referred to the Musculoskeletal Central Intake and Assessment Centre. The patients were assessed by the orthopedic surgeons and APP, of whom 195 (19 %) were considered candidates for hip or knee surgery and who were then screened for study eligibility. Of these, 69 (35 %) were enrolled in the study. Among potential participants excluded, 84 (67 %) were not interested and 42 (33 %) did not meet eligibility criteria for several reasons shown in Fig. 1. The retention rate for both intervention phase and study completion was 81 %. Data completion ranged from 80 % (95 % CI: 68 – 88 %) for frailty index to 85 % (95 % CI: 73 – 93 %) for Oxford Hip Score at the 6 months postoperative follow-up visit. The reasons for incomplete data were study withdrawal 8 (57 %), missed appointment 5 (36 %), and unknown 1 (7 %).

**Table 2:** Feasibility outcomes.

Outcome	Evaluation metric	Estimate	Criteria for success
Recruitment	% patients assessed out of all referred surgical patients	n (%) 195 (19)	≥80 %
	% patients enrolled out of all patients assessed for eligibility	69 (35)	≥80 %
Retention	% participants who completed intervention phase and the study	n (%) 56 (81)	≥80 %
Data completion	% participants with complete data for the following outcomes:	% (95 % CI)	≥80 %
	SPPB	81 (70 – 89)	≥80 %
	Oxford hip score	85 (73 – 93)	
	Fit frailty index	80 (68 – 88)	
Participants adherence	Intervention components		
	Exercise Strength: average number of days/week	Mean (95 % CI) 4.0 (3.2 – 4.7)	≥2 days
	Aerobic: average number of mins/week	92.1 (63.5 – 120.7)	≥150 mins/week
	Balance: average number of days/week	2.9 (2.2 – 3.7)	≥2 days
	Flexibility: average number of days/week	3.4 (2.8 – 4.0)	≥2 days
	Protein supplement % daily protein supplements consumed per month	% (95 % CI) 66.5 (54.6 – 78.4)	≥80 %
	Vitamin D % daily Vitamin D consumed per month	% (95 % CI) 82.3 (73.4 – 91.5)	≥80 %
	Medication review % participants who received medication review % participants who implemented recommendations	% (95 % CI) 86.2 (67.5 – 95.0) 41.4 (24.6 – 60.4)	≥80 %

n, number; %, percentage; CI, confidence interval; SPPB, Short Physical Performance Battery.

The average intervention period for participants was 5.2 months (SD: 3.0). The time between enrolment and surgery were similar between the intervention and control groups. The mean self-reported adherence for physical exercise types were strength 4 days (95 % CI: 3 – 5 days/week), aerobic 92 mins (95 % CI: 64 – 121 mins/week), balance 3 days (95 % CI: 2 – 4 days/week), flexibility 3 days (95 % CI: 3 – 4 days/week). On average, participants consumed 67 % (95 % CI: 55 – 78 %) of protein supplements and 82 % (95 % CI: 73 – 92 %) of Vitamin D per month. During the routine adherence check-ins, some participants reported concerns with the limited options of protein supplement flavours and fear of gaining weight by consuming the supplement daily. For medication review, 86 % (95 % CI: 68 – 95 %) received a consultation and 41 % (95 % CI: 25 – 60 %) met with their family physician who reviewed the study pharmacist's recommendations.

### 3.3 Preliminary effectiveness outcomes

Table 3 shows the effects of the intervention on Oxford Hip Score, Oxford Knee Score, SPPB, frailty index and EQ-5D-3L health-related quality of life at the three follow-up timepoints. At the 6 months postoperative visit, the intervention group had a significantly higher score, 8.78 (95 % CI: 0.40 – 17.16) for knee replacement patients. There were also a clinically relevant but not statistically significant change in the Oxford Knee Score at 6-weeks post-surgery, 9.11 (95 % CI: −2.66 – 20.87). Other outcomes including the frailty score, −0.04 (95 % CI: −0.10 – 0.01) at 6 months post-surgery and health-related quality of life, 0.04 (−0.04 – 0.12) at 6-weeks postoperatively showed clinically meaningful differences in favor of the intervention. The results of the complete case analyses (Supplementary Table 1) were similar to those of the intention-to-treat analyses.

**Table 3:** Clinical Outcomes.

Outcome	Mean (SD)		Mean difference (95 % CI)
	Control	Intervention	
**Oxford Hip Score**			
1 week preoperative	17.73 (7.14)	16.50 (7.30)	−1.17 (−5.22 – 2.87)
6 weeks postoperative	36.73 (7.80)	37.33 (7.99)	1.44 (−3.40 – 6.28)
6 months postoperative	41.39 (7.11)	42.96 (4.74)	1.58 (−1.84 – 5.01)
**Oxford Knee Score**			
1 week preoperative	18.28 (8.56)	21.25 (14.97)	4.90 (−6.94 – 16.75)
6 weeks postoperative	30.00 (9.38)	39.33 (8.14)	9.11 (−2.66 – 20.87)
6 months postoperative	37.85 (6.67)	42.00 (2.65)	8.78 (0.40 – 17.16)*
**SPPB**			
1 week preoperative	6.87 (2.67)	6.73 (2.79)	−0.06 (−1.44 – 1.32)
6 weeks postoperative	7.46 (2.50)	7.25 (3.01)	−0.03 (−1.47 – 1.42)
6 months postoperative	9.00 (2.302)	8.61 (2.45)	−0.38 (−1.57 – 0.82)
**Frailty index**			
1 week preoperative	0.33 (0.12)	0.33 (0.08)	0.02 (−0.04 – 0.07)
6 weeks postoperative	0.22 (0.09)	0.22 (0.09)	−0.01 (−0.06 – 0.04)
6 months postoperative	0.23 (0.11)	0.18 (0.10)	−0.04 (−0.10 – 0.01)
**EQ-5D-3L**			
1 week preoperative	0.62 (0.16)	0.57 (0.19)	−0.04 (−0.13 – 0.05)
6 weeks postoperative	0.82 (0.16)	0.86 (0.13)	0.04 (−0.04 – 0.12)
6 months postoperative	0.82 (0.16)	0.85 (0.17)	0.02 (−0.06 – 0.11)

⁎ p-value = 0.04; SD, standard deviation; CI, confidence interval; EQ-5D-3L, European Quality of Life, 5 Dimension 3 Levels; SPPB, Short Physical Performance Battery.

**Supplementary Table 1:** Clinical Outcomes (complete case analyses).

Outcome	Mean (SD)		Mean difference (95 % CI)
	Control	Intervention	
**Oxford Hip Score**			
1 week preoperative	19.05 (6.65)	16.11(7.72)	−2.94 (−7.46 – 1.57)
6 weeks postoperative	36.35 (7.82)	36.63 (8.09)	0.28 (−4.71 – 5.28)
6 months postoperative	42.70 (4.86)	43.05 (5.10)	0.35 (−2.77 – 3.48)
**Oxford Knee Score**			
1 week preoperative	17.20 (8.53)	14.33 (7.02)	−2.87 (−14.40 – 8.67)
6 weeks postoperative	27.80 (8.58)	39.33 (8.14)	11.53 (−0.55 – 23.61)
6 months postoperative	35.60 (6.58)	42.00 (2.65)	6.4 (−1.59 – 14.40)
**SPPB**			
1 week preoperative	7.20 (2.66)	6.91 (2.81)	−0.29 (−1.86 – 1.27)
6 weeks postoperative	7.48 (2.63)	7.45 (2.81)	−0.03 (−1.58 – 1.53)
6 months postoperative	9.32 (2.29)	8.73 (2.47)	−0.59 (−1.95 – 0.77)
**Frailty index**			
1 week preoperative	0.30 (0.11)	0.33 (0.09)	0.02 (−0.04 – 0.08)
6 weeks postoperative	0.22 (0.09)	0.22 (0.09)	−0.003 (−0.06 – 0.05)
6 months postoperative	0.21 (0.08)	0.18 (0.10)	−0.03 (−0.09 – 0.02)
**EQ-5D-3L**			
1 week preoperative	0.63 (0.16)	0.58 (0.20)	−0.05 (−0.15 – 0.05)
6 weeks postoperative	0.83 (0.15)	0.87 (0.13)	0.04 (−0.04 – 0.12)
6 months postoperative	0.83 (0.16)	0.87 (0.14)	0.04 (−0.05 – 0.13)

SD, standard deviation; CI, confidence interval; EQ-5D-3L, European Quality of Life, 5 Dimension 3 Levels;.

SPPB, Short Physical Performance Battery.

### 3.4 Adverse events

In total, there were 83 adverse events reported – 40 (48 %) in the control vs 43 (52 %) in the intervention arm. The adverse events included pain 21 (25 %), falls 12 (14 %), diarrhea 5 (6 %), dizziness 3 (4 %), fractures 2 (2 %), and others 40 (48 %). The events occurred in 44 participants – 23 participants (52 %) in the control and 21 participants (48 %) in the intervention arm. Most of the adverse events 58 (70 %) were unrelated to the intervention. The fractures reported were not associated with the intervention and only 2 (17 %) of the falls and 9 (43 %) of pain were either possibly or probably linked to the intervention. Six serious adverse events were reported but they were not related to the intervention.

## 4 Discussion

This study provides evidence of feasibility on participant retention, data completion, and participant adherence to intervention components including exercise, Vitamin D supplement intake, and medication review consultation in the Geras FitJoints multimodal prehabilitation program for older adults with frailty awaiting total hip or knee replacement. Participant recruitment, adherence to protein supplements and aerobic exercise as well implementation of medication review recommendations did not meet the prespecified criteria for feasibility success. The evaluation of clinical outcomes showed that the Oxford Knee Score at 6-months post surgery was significantly better than in the intervention group. Also, there were signals of benefits for physical performance, frailty status and quality of life postoperatively. Only a few self-reported adverse events were potentially attributable to the intervention.

Recruitment of participants is critical to the success of any trial; as such, it is a key indicator of feasibility of reaching planned sample size for future larger trials [45]. The recruitment rate did not meet the targeted progression cut-off of 80 % due to reasons such as patients not having the minimum 3-month wait time before surgery, being below 60 years, and unavailable when contacted. In retrospect, we consider the blanket criterion for success of 80 % applied to most feasibility outcomes in this study too high for the recruitment measure. A specific target informed by previous studies in similar populations and the current study context would have been more realistic. Recruitment rates from previous studies ranged from 34 – 79 % [20,21,22], averaging around 60 %. Based on the existing data, our study's recruitment rate of 35 % is at the low end. Considering the long duration of recruitment and low number of potential participants screened to attain this rate, achieving higher recruitment rates for a larger sample size in future trials would require some modifications of the current study design including recruiting from multiple sources.

Generally, recruitment of older adults living with frailty is challenging due to poor health and mobility issues that may limit their inclusion or participation in studies [46]. Additionally, the patient group for the study may not be considered for surgery due to the high risk for unfavourable outcomes [47], thus affecting recruitment. More flexibility with time and space of study [46], use of virtual platforms for intervention delivery [48], and engagement of family caregivers [49] could accommodate for the health and mobility barriers. Also, close monitoring of the recruitment process and a flexible protocol could allow for the adjustment of strategies to reach required numbers. Overall, the insights gained from this study could be used to better inform recruitment in future studies.

As a definite positive finding, the retention target was achieved and is comparable to previous trials [20,21,22]. With a follow-up duration of 9 – 16 months, the study's retention rate of 81 % for both completion of intervention phase and the entire study is encouraging for future trials. The data completion measure was equally comparable to retention, with few participants having incomplete outcome data due to missed appointments or withdrawal.

Adherence to all exercise components was optimal except for aerobic exercises. On average, participants performed 38 % below the recommended 150 mins/week of moderate to vigorous aerobic exercise per the guideline used in the study [39]. Although higher levels of exercise adherence have been reported in systematic reviews in the general orthopedic surgical patient population [17,18], it is not clear if the exercises were aerobic. We noted during adherence tracking that participants reported difficulty completing aerobic exercises due to pain given that it required a longer duration to perform. It is possible that the exercises may have been challenging for this patient population since the prescription was based on exercise guidelines designed for health older adults.

Aerobic exercise was the only exercise type where frequency and time were reported while only frequency was reported for other components in this trial. The evidence regarding the FITT (Frequency, Intensity, Time and Type) [49] of exercise that is most effective for frailty prevention and management is inconclusive [50]. More studies are required to demonstrate the FITT of exercises that is feasible and effective for older adults with frailty. This will not only help in the prescription, assessment, and reporting of physical exercise but will potentially improve adherence in future studies. Given that study participants had the choice of where, when, and how they exercised, stepping up accountability through supervised group exercise sessions could potentially improve exercise adherence as shown in our recent study [48]. Further, future trials should consider the use of objective measures of physical activity such as accelerometers for more reliable and valid measurements [51].

Nutrition adherence was met for Vitamin D but not for protein supplements, albeit the results fall within the range (50 – 100 %) reported in recent systematic reviews [52,53]. The observed lower compliance with protein supplements (67 %) compared to Vitamin D supplements (82 %) may be attributed to participants concerns that were noted during routine adherence check-in, which included reports of limited options of the flavours of protein supplement provided and fear of gaining weight with the recommended daily protein supplements. The average body mass of the study participants tended towards overweight. Excess weight could impair post-surgical recovery [54,55] and patients who have obesity are typically advised to lose weight prior to surgery for better outcomes [54]. We did not assess baseline dietary protein intake to provide specific recommendation for protein supplements. Tailoring prescribed nutrition supplements to participant's current dietary needs, clinical profile, and preferences could improve adherence in future studies.

Adherence to the implementation of medication review recommendations was low. Given the high prevalence of polypharmacy (currently taking ≥ 5 or more drugs) in the study (75 %) which increases the risk of taking potentially inappropriate medications and drug-drug interactions [56], review for potential deprescribing, medication optimization, and harm reduction is needed [57]. The reasons for low adherence to the medication recommendations were not explored in our study, as such, it presents an area of investigation for future of studies in order to enhance the success of this intervention component.

The exploratory analyses of clinical outcomes showed clinically meaningful [58] and statistically significant difference for knee pain at 6 months postoperative timepoint, and only clinically meaningful changes for frailty [59] and health-related quality of life [60]. Although, our study was not powered for statistical tests of efficacy, these results provide indications of benefits that could be demonstrated in larger definitive trials. Recent meta-analyses in the general arthroplasty patient population [17,18] suggests varying effects for both preoperative and postoperative outcomes. Therefore, future adequately powered trials in older adults with frailty are required to provide firm conclusions about the efficacy of the prehabilitation intervention in the short and long term perioperatively. Overall, the data on adverse events suggests that the intervention is safe with minimal risks to participants.

### 4.1 Strengths and limitations

This study addressed the limitations of previous studies including generalizability (it included patients regardless of the type of joint surgery or gender), longer duration of intervention, multimodal intervention, and assessment of short- and long-term outcomes. Previous trials have evaluated prehabilitation interventions in total hip replacement patients with frailty [20,21,22], while this study included both hip and knee replacement patients. In addition, the multimodal intervention in this study consisted of several components that are important for improving outcomes in frail patients (i.e., exercise, protein and vitamin D supplementation, medication review), whereas other prehabilitation interventions have included fewer components (e.g., exercise only, or exercise plus nutrition) [20,21,22]. However, the results of this study should be interpreted in the context of the limitations. First, there were no measures to validate participant's self-reported adherence to intervention components, so there is a possibility of under-reporting or over-reporting of outcomes. Second, the trial was single-blinded; as such, participants behaviour and responses may have been influenced by the knowledge of their group assignment. Third, the efficacy analyses were only exploratory as our study was not sufficiently powered to detect differences in effects.

## 5 Conclusion

Our study provides valuable preliminary data on feasibility and efficacy of prehabilitation in older adults with frailty awaiting joint replacement with a clear positive effect on patients undergoing total knee replacement at 6-months postoperatively. While retention, data completion, adherence to some intervention components were optimal with respect to prespecified criteria, some aspects of the study design require modifications for better outcomes. We recommend increasing study recruitment sites, adapting nutrition recommendations to participants’ usual intake, needs and preferences, exploring reasons for low adherence to the implementation of medication review recommendation, utilising FITT principle for exercise prescription and reporting as well as increasing exercise accountability for better adherence. Larger trials powered to detect clinically significant differences are required for definitive guidance on effectiveness of prehabilitation in this patient population.

## Abbreviations

RCT, Randomized controlled trial; SD, Standard deviation, CI, Confidence interval; OA, osteoarthritis; CONSORT, Consolidated Standards of Reporting Trials; MSK CIAC, Musculoskeletal Central Intake and Assessment Centre; RJAP, Regional Joint Assessment Program; APP, Advanced Practice Physiotherapists; RA, Research assistant, TIDieR, Template for Intervention Description and Replication; SPPB, Short Physical Performance Battery; EQ-5D-3L, European Quality of Life 5-dimension 3-level; REDCap, Research Data Capture; FITT, Frequency, Intensity, Type and Time.

**Trial Funding**: Hamilton Academic Health Science Organization and Hamilton Health Sciences Research Strategic Initiatives RFA. Abbott Nutrition provided protein supplements for the study. The trial funders had no role in the study design, data collection, analysis, and interpretation, writing of report and decision to submit the manuscript for publication.

## Authors’ contribution

Conceptualization: AM, MW, JDB, VA, BD, JDA, GI, CK, AL, SM, DA, SA, BL, AP, DW and AP. Formal analysis: CO, GI, LT and AP. Investigation: AP, AM, MW, JDB, VA, BD, JDA, DP, GI, CK, AL, SM, DA, SA, GH BL, and AP. Writing - original draft: CO. Writing - review & editing: AM, MW, JDB, VA, BD, JDA, DP, LT, GI, CK, AL, SM, DA, SA, GH, BL, AP, DW and AP. All authors approved the final version of the manuscript.

## Ethics approval and consent to participate

The study was approved by the Hamilton Integrated Research Ethics Board (file #2017–1565). All participants provided written informed consent prior to randomization.

## Competing interests

DA reports research funding: Weston Foundation and Nestlé Health Sciences; consulting: Cinclus Pharma and Phathom Pharma; speaker honorarium: Fresenius Kabi and Takeda Canada, Amgen; industry: Co-founder A.I. VALI; not-for-profit: Board member, Canadian Digestive Health Foundation, Treasurer and Board member, International Working Group for the Classification of Oesophagitis. JL reports personal fees from Research Institute of St. Joe's, grants from Canadian Institutes of Health Research (CIHR), and grants from United States Deprescribing Network (USDeN), outside the submitted work. SM reports grants from Daniels Foundation, grants from CIHR, personal fees from Unity Health Toronto, and other from Unity Health Toronto, Providence Health Care, during the conduct of the study. All other authors (CO, AN, JDA, SAA, VA, JDB, GH, GI, CK, PH, AL, JR, AP, DP, LT, MW, AP) declare that they have no competing interests.
